# Signal-carrying speckle in optical coherence tomography: a methodological review on biomedical applications

**DOI:** 10.1117/1.JBO.27.3.030901

**Published:** 2022-03-14

**Authors:** Vania B. Silva, Danilo Andrade De Jesus, Stefan Klein, Theo van Walsum, João Cardoso, Luisa Sánchez Brea, Pedro G. Vaz

**Affiliations:** a University of Coimbra, Laboratory for Instrumentation, Biomedical Engineering and Radiation Physics (LIBPhys-UC), Department of Physics, Coimbra, Portugal; b University Medical Center Rotterdam, Department of Radiology and Nuclear Medicine, Erasmus MC, Biomedical Imaging Group Rotterdam, Rotterdam, The Netherlands

**Keywords:** speckle, image processing, image analysis, imaging coherence, tomography

## Abstract

**Significance:**

Speckle has historically been considered a source of noise in coherent light imaging. However, a number of works in optical coherence tomography (OCT) imaging have shown that speckle patterns may contain relevant information regarding subresolution and structural properties of the tissues from which it is originated.

**Aim:**

The objective of this work is to provide a comprehensive overview of the methods developed for retrieving speckle information in biomedical OCT applications.

**Approach:**

PubMed and Scopus databases were used to perform a systematic review on studies published until December 9, 2021. From 146 screened studies, 40 were eligible for this review.

**Results:**

The studies were clustered according to the nature of their analysis, namely static or dynamic, and all features were described and analyzed. The results show that features retrieved from speckle can be used successfully in different applications, such as classification and segmentation. However, the results also show that speckle analysis is highly application-dependant, and the best approach varies between applications.

**Conclusions:**

Several of the reviewed analyses were only performed in a theoretical context or using phantoms, showing that signal-carrying speckle analysis in OCT imaging is still in its early stage, and further work is needed to validate its applicability and reproducibility in a clinical context.

## 1 Introduction

Optical coherence tomography (OCT) is an optical imaging modality based on low-coherence interferometry. It is a noninvasive technique that provides *in vivo* cross-sectional images of microscopic structures with high spatial and temporal resolutions, making it an appealing technique for multiple areas in preclinical and clinical research.^1^

**Fig. 1:**
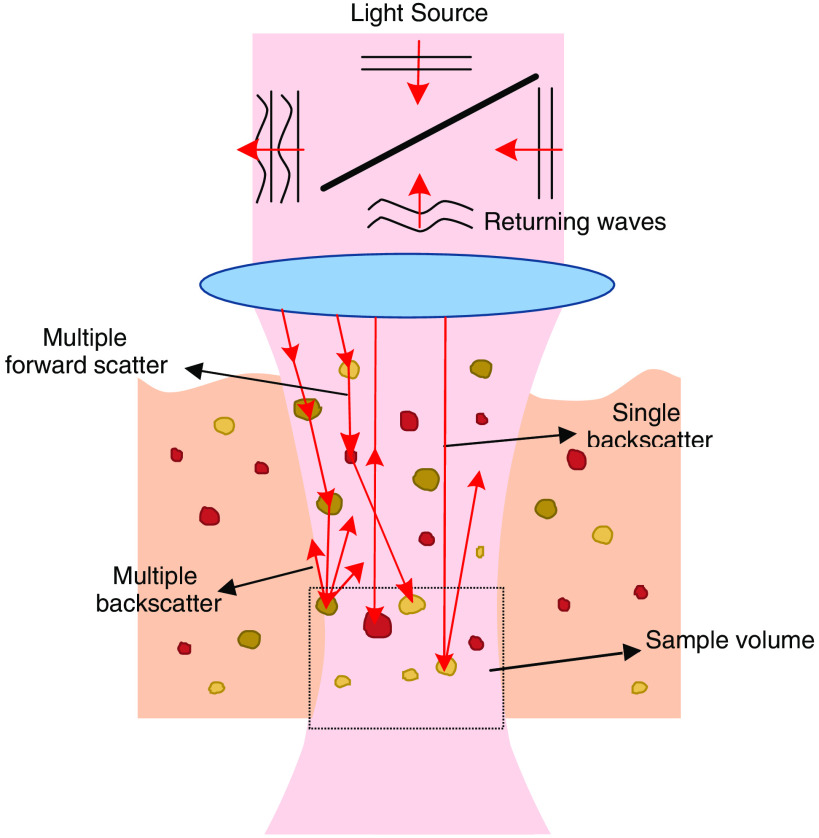
Process of speckle formation in OCT. Speckle patterns result from coherent superposition of multiple backscattered and multiple forwardscattering waves from particles in the sample volume.

In OCT imaging, the tissue is scanned by an optical beam, and most of the light is either refracted or scattered. The incident light travels through different optical paths, with different lengths, until it reaches the image plane. The light intensity at each point of the plane results from destructive/constructive interference of all light waves at that single point. This phenomenon, shown in Fig. 1, creates granular patterns, known as speckle patterns. Speckle appears everywhere when an optically rough surface is illuminated with coherent light, making it common to all coherent imaging modalities.^2^

Schmitt et al.^3^ were among the first to discuss the OCT speckle origin, distinguishing between two types of mechanisms that cause the formation of speckle patterns on a cross-sectional image: multiple backscattering of the light beam, and delays caused by multiple forward scattering. Speckle patterns are then influenced by different parameters, such as the properties of the light source, the propagating beam, the aperture of the detector, and the inner properties and structural organization of the tissues.^4^^,^^5^ Since the sample properties have an influence on speckle formation, it may contain relevant information regarding subresolution and structural properties of the tissues from which it originated.^6^ In fact, in the work presented by Schmitt et al.,^3^ speckle patterns in OCT are already mentioned as having a dual role, both as a source of noise, signal-degrading speckle, and as a carrier of information, signal-carrying speckle. This indicates that, besides the granular noise observed in OCT raw images, the imaged speckle also carries information and this information may be used to characterize the imaged tissue.^7^^,^^8^

A large number of works have been focused on speckle as a source of noise in OCT imaging, as it reduces the image quality and contrast, making boundaries between tissues less distinguishable. Because of this, methods to suppress and reduce speckle have been developed, including filtering,^9^ averaging,^10^ or wavelet processing techniques.^11^ Since most of the works in the literature focus on signal-degrading speckle, the information regarding signal-carrying speckle analysis is diffuse and sometimes abstruse. Thus, this review intends to provide a comprehensive overview of the different methods used to retrieve information from OCT speckle in biomedical applications.

The remainder of this paper is structured as follows: Sec. 1 presents the introduction; Sec. 2 presents the literature search criteria; Sec. 3 presents the signal processing methods used for analysing the OCT signal-carrying speckle. Discussion of some of the most relevant approaches is presented in Sec. 4. Finally, the conclusions are presented in Sec. 5. A table summarizing the reviewed works is given in Appendix A, and a short theoretical mathematical description of light speckle is given in Appendix B.

## 2 Methods

**Fig. 2:**
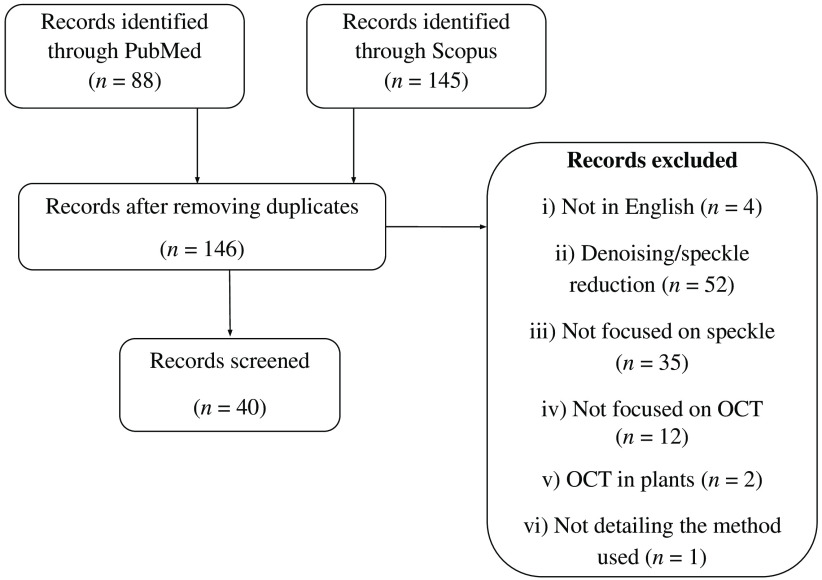
Flowchart of the records selection.

The literature search was conducted in two databases on December 9, 2021. PubMed was chosen for being one of the largest databases in the medical field, and Scopus for combining articles from both medical and technical fields. The search query used was: “Optical Coherence Tomography” AND speckle AND (statistics OR statistical) NOT flowgraphy. After duplicate removal, the total number of articles obtained was 146. These articles were screened and narrowed down to 40. The applied exclusion criteria were: (i) not written in English, (ii) focusing on denoising/speckle reduction, (iii) not focusing on speckle, (iv) not focusing on OCT, (v) OCT used in plants, and (vi) not detailing the method used. The number of articles excluded by each criterion is detailed in Fig. 2. The remaining 40 articles were then reviewed.

The data extracted from each article were the implemented method, the OCT technique used, the light-source wavelength (for non-theoretical studies), the biomedical application, and performance metrics related to the application, when provided. This information is reported in Table 1, in Appendix A.

## 3 Results

**Fig. 3:**
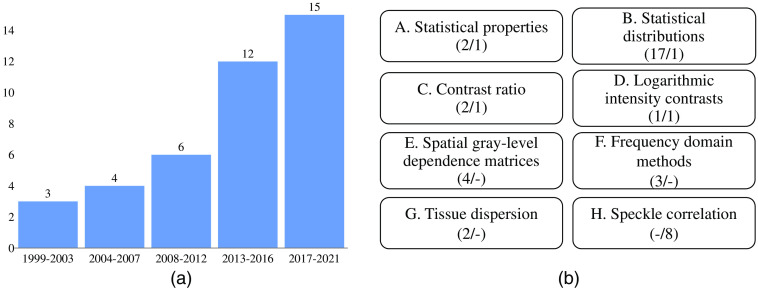
Overview of the results. (a) Distribution of the articles, grouped by publication year. (b) The number of articles included for each method performing a static (s) and dynamic (d) analysis is included as (s/d).

Figure 3(a) shows the distribution of the articles included in the review grouped by year. The results show a growing interest in the analysis of signal-carrying speckle in OCT imaging over the last two decades.

Figure 3(b) clusters the reviewed articles by technique, depicting the organization of Sec. 3 where the most represented technique corresponds to “statistical distributions.” Since speckle pattern analysis can either provide static or dynamic information about the imaged tissue, depending on whether the scatterers are stationary or in motion between consecutive image acquisitions, the articles were subsequently divided according to this classification.

### 3.1 Statistical Properties

Local moment-based statistical properties of OCT signal intensity have been used for inferring speckle characteristics. These properties have proven to be useful in classification tasks, allowing to discriminate between different types of tissue.

#### 3.1.1 Static analysis

Roy et al.^12^ used the mean (μ), standard deviation (σ), kurtosis (κ), skewness (ν), and an estimate of optical attenuation and signal confidence measures to detect plaques’s susceptibility to rupture using intravascular OCT, with the objective of assessing atherosclerosis. The features proved to have high performance in the identification of such tissues using a random forest predictive model (area under the receiver operating characteristics curve of 0.9676).

Wang et al.^13^ implemented a model for the detection of soft tissue sarcomas in OCT images of *ex vivo* human tissues, also based on speckle statistical properties. Specifically, the standard deviation of the signal fluctuations (speckles) of a single axial line (A-scan) was used. The statistical analysis (Student’s t-test) of the standard deviation showed it is effective for comparing normal fat tissue and soft tissue sarcoma (p value<0.01).

#### 3.1.2 Dynamic speckle analysis

**Fig. 4:**
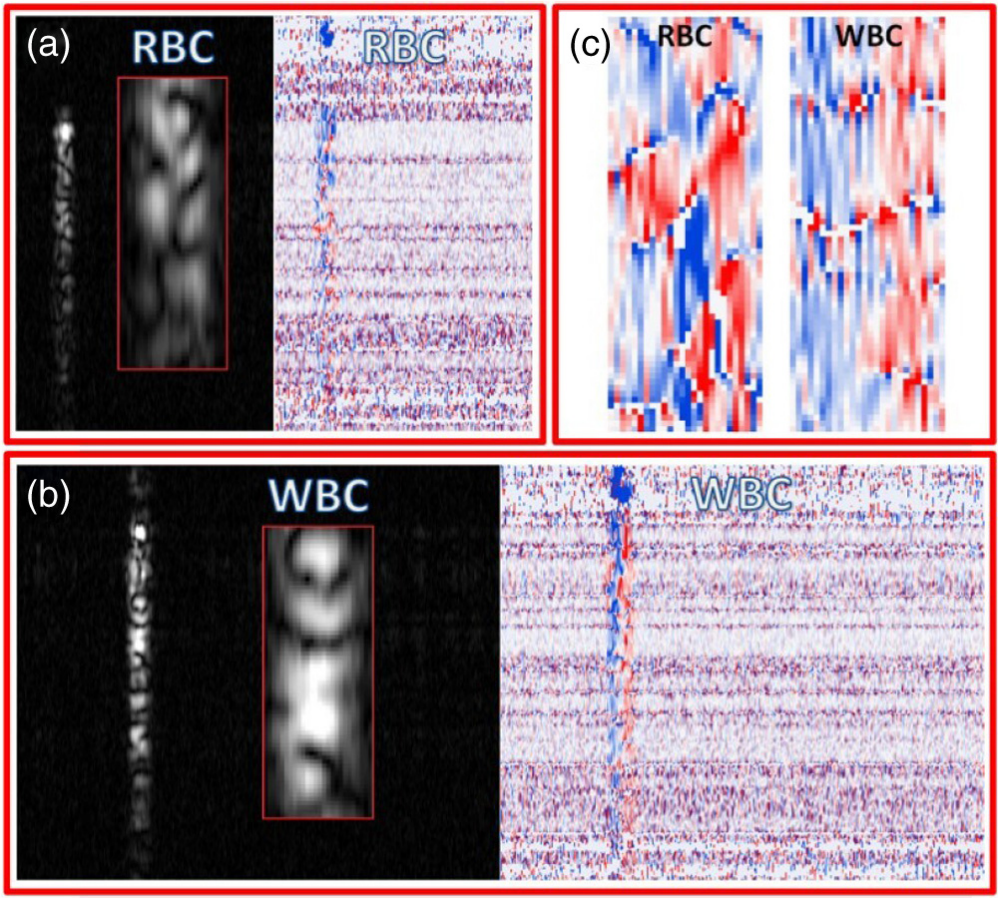
Intensity and phase-change images originated from modulation signal of: (a) erythrocytes (red blood cells, RBC) and (b) leukocytes (white blood cells, WBC). (c) An enlarged subsection of RBC and WBC phase-change images, containing entire signals transversely. Reproduced from Ossowski et al.^14^ with the authors’ permission.

Ossowski et al.^14^ used statistical properties of the OCT speckle to infer the dynamic properties of blood samples. Specifically, they used the mean horizontal and vertical speckle sizes, calculated from intensity data, and the sum of standard deviations of selected windows, calculated from phase data. These three statistical parameters were computed in OCT images of blood samples [Figs. 4(a) and 4(b)], enabling a visual distinction between the signal modulation from erythrocytes and leukocytes as shown in Fig. 4. No statistical tests were performed to assess the discriminating power of these metrics.

### 3.2 Statistical Distributions

In Appendix B, the theoretical distributions for conventional laser speckle imaging complex amplitude (Gaussian distribution) and intensity (exponential distribution) are presented. In laser speckle, the intensity pattern is often directly observed, while in OCT the modulus and phase are detected.^7^ By applying the required transformation from the speckle intensity to the speckle modulus probability density function (PDF), we arrive at the Rayleigh distribution.

These distributions have a clear physical meaning, but they are only applicable to an ideal, fully developed, speckle pattern. This type of speckle pattern is formed when the amplitude and the phase of the speckle field are statistically independent variables and when the phases are uniformly distributed in the range of (−π, π). This may not always be the case in real world applications due to several reasons, among others: the presence of dynamic scatterers, the small optical roughness when compared with the light wavelength, or the low scatterers’ concentration of certain regions of the reflections surfaces.

In these cases, speckle formation could be difficult to model. Therefore, different PDFs have been proposed to describe the speckle statistics in real world applications of OCT imaging. The parameters of these distributions are expected to change according to the light source properties and dimension/organization of the scatterers in the sample, thus providing information about the tissue properties. Given the different notations and formulations in the literature, a coherent mathematical notation of the proposed models is provided.

This section is organized as follows: in Sec. 3.2.1, Rayleigh distribution is presented. This is the fundamental distribution used to represent fully developed speckle patterns. In Sec. 3.2.2, the Gamma and generalized Gamma (GG) distributions are detailed. From these two, the remaining distributions, presented in Sec. 3.2.3, can be derived, including K-distribution, Weibull, Nakagami, Rician, three-parameter Rayleigh, Lognormal, and Burr type XII. Next, a nonparametric approach is presented in Sec. 3.2.4. Finally, Sec. 3.2.5 details the distributions that have been applied in a dynamic speckle analysis.

#### 3.2.1 Fundamental distribution

The Rayleigh distribution is a one-parameter distribution, used to model fully developed OCT speckle patterns. The application of this model is valid when the signal arises from multiple scatterers within the resolution of the system,^15^ and the light complex field amplitude is represented by circular Gaussian statistics, i.e., a fully developed speckle pattern.^16^ Its PDF is given as

$$p_{\mathrm{RL}}(A;a)=\frac{A}{a^{2}}e^{\left(-\frac{A^{2}}{2a^{2}}\right)},$$ (1)

where a is the scale parameter.

Almasian et al.^17^ experimentally verified the goodness of fit of the Rayleigh PDF for modeling speckle amplitude using controlled samples of silica microspheres suspended in water. They proved that OCT amplitude distribution for homogeneous samples can be described by a Rayleigh distribution for images with low optical depth (coefficient of determination, $R^{2}\approx 0.98$). Also, assuming a Rayleigh distribution, expressions were analytically derived for speckle signal mean amplitude and variance in terms of sample scattering coefficient and backscattering coefficient.

In addition, Ossowski et al.^18^ performed a study using a realistic simulation method based on Maxwell’s equations and an experimental polydimethylsiloxane and ${\mathrm{TiO}}_{2}$ phantom.^19^ This work provided evidence that the speckle amplitude related to an homogeneous scattering region of the phantom follows a Rayleigh distribution and confirm the agreement between the simulation and experimental results.

#### 3.2.2 Gamma distributions

The Gamma distribution is a two-parameter distribution. Its PDF belongs to a family of PDFs with two degrees of freedom, and is defined as

$$p_{G}(A;a,d)=\frac{A^{d-1}e^{-A/a}}{a^{d}\Gamma (d)}\;\text{for }a,d>0,$$ (2)

where d is the shape parameter, a is the scale parameter, and Γ represents the Gamma function.^20^

Kirillin et al.^21^ developed a Monte Carlo model for speckle statistics simulation of OCT data and validated the model using a phantom. Also, they demonstrated by visual inspection that the Gamma distribution was a good fit for both phantom and the previously simulated data. The scale parameter, a, showed an increase with the increase of scatterers concentration, whereas the shape parameter, d, presented a concentration-independent behavior, but it has been related to the effective scatter number density in other work using high-frequency ultrasound.^22^

More recently, Niemczyk et al.^23^ used the Gamma distribution to model speckle from corneal OCT data in porcine eyes. Both Gamma parameters showed a statistically significant relation with intraocular pressure (IOP) (p value<0.001, ANOVA test).

The GG distribution is a three-parameter generalization of the Gamma distribution, with a PDF given as

$$p_{\mathrm{GG}}(A;a,d,p)=\frac{pA^{d-1}}{a^{d}\Gamma (d/p)}e^{-{\left(A/a\right)}^{p}}\;\text{for }p>0,$$ (3)

where d and p are shape parameters, and a is the scale parameter. To obtain the Gamma PDF [Eq. (2)], p must be set to 1. Special cases of GG include the previously presented Rayleigh [Eq. (1)], by setting the parameters to $p_{\mathrm{GG}}(A;a\sqrt{2},2,2),a>0$.

**Fig. 5:**
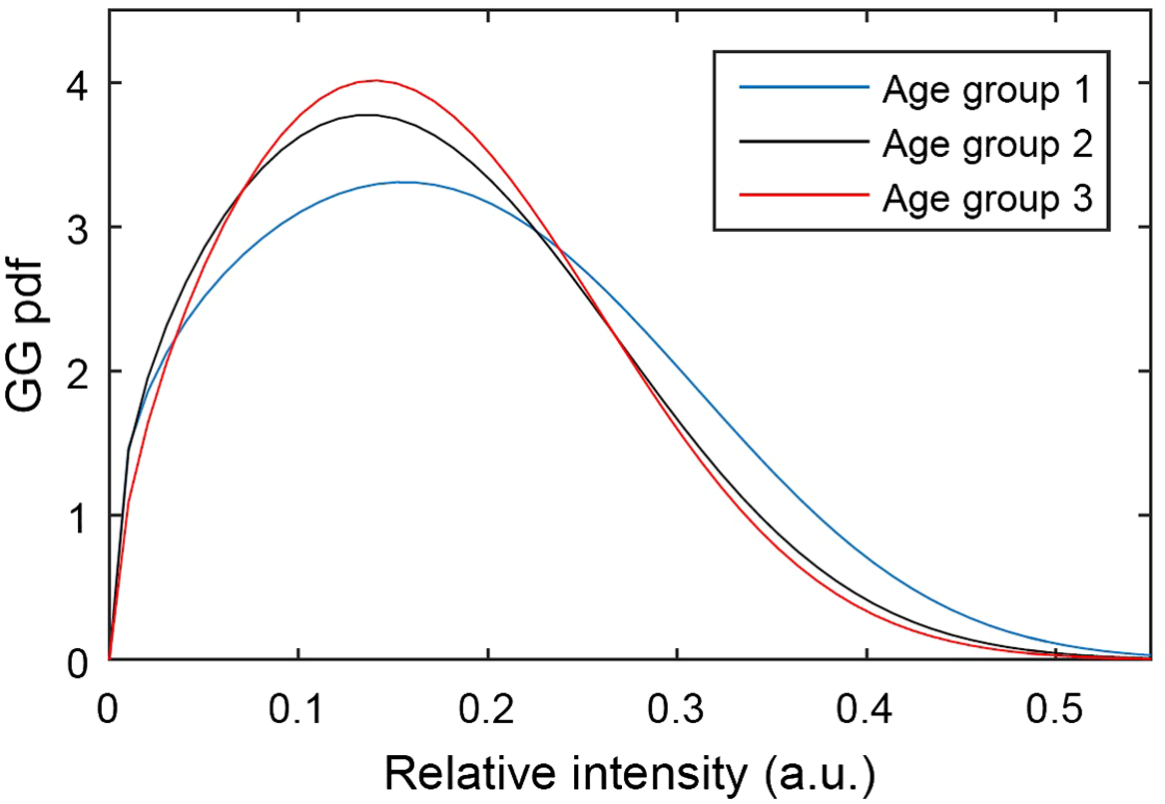
PDF of the GG distribution for three different age groups, where age group 1 is the youngest and age group 3, the oldest. Reproduced from Jesus et al.^4^ with the authors’ permission.

The GG distribution was used by Jesus et al.^4^^,^^24^^,^^25^ and Iskander et al.^26^ to model corneal OCT data. Jesus et al.^4^^,^^25^ applied the GG distribution to healthy subjects divided in three age groups (24.4±0.5, 31.3±4.6; 61.2±8.4 years), as shown in Fig. 5. The goal was to study variations of the distribution parameters among groups. A significant statistical difference, (p value<0.05, Kruskal–Wallis test) was observed for all three parameters. For an increase in age, the scale (a) and shape (p) parameters decreased, while the parameter relation d/p increased, resulting in a narrow distribution. These microstructural changes are related with the stiffer corneal tissue presented by older subjects.

In a later study,^24^ Jesus et al. analyzed the parameters’ relation with microstructural corneal properties. Significant correlation (p value<0.001) was found between both the scale parameter (a) and the ratio of the shape parameters (d/p) with IOP, where the scale parameter decreases when the IOP increases. A possible physical explanation for this phenomenon is the influence of IOP on the decrease of interlamellar gaps (absence of collagen), as studied by Wu et al.,^27^ resulting in a more compacted tissue. This shifts the speckle distribution toward lower values, similarly to the age effect.

Moreover, Danielewska et al.^28^ ascertain the influence of IOP in untreated and crosslinked rabbit eyes. The GG distribution achieved the best goodness of fit against Rayleigh, Weibull, Nakagami, and Gamma distributions, considering the mean squared error between the fitted PDF and the kernel density estimator (KDE). Both the scale and ratio parameters presented significant statistical differences with respect to increasing IOP in anterior and central corneal stroma. In addition, the GG distribution shape parameter (p) was also statistically significant when comparing untreated and crosslinked for anterior, central, and posterior corneal stroma.

Iskander et al.,^26^ used the GG to model information from the microstructure of the cornea to differentiate glaucoma suspects, glaucoma patients, and healthy controls. ANOVA tests showed that the scale parameter, a, was correlated with the shape parameter, p, and the relation between these two parameters was statistically significantly different between the three study groups (p value<0.0001, Fisher’s test). Finally, Seevaratnam et al.^1^ used the GG distribution to investigate the effect of temperature variation in tissue phantoms. The scale parameter, a, showed a linear increase with the increase of the tissue temperature. The correlation between a and the temperature was statistically significant ($p\text{ value}=7.9\times 10^{-6}$, Student’s t-test). An increase in temperature results in higher scatterers motion amplitudes and, consequently, in a wide range of amplitude values.

#### 3.2.3 Gamma derived distributions

The K-distribution is a three-parameter distribution, used to model cases where a small number of scatterers are present in the sample,^4^ resulting in partially or nonfully developed speckle patterns. Its PDF can be written as

$$p_{K}(A;\upsilon ,\varphi ,L)=\frac{2\xi^{(\beta +1)/2}A^{(\beta -1)/2}}{\Gamma (L)\Gamma (\varphi )}K_{\varphi -L}(2\sqrt{\xi A}),$$ (4)

where β=L+φ−1, ξ=Lφ/μ, $K_{\alpha }$ is a modified Bessel function of the second kind of order α. This distribution is the combination of two gamma distributions, one with mean 1 and shape parameter φ and the other with mean υ and shape parameter L.

The K-distribution was tested by Jesus et al.^4^ for corneal OCT speckle intensity characterization against other distributions. However, using a Kolmogorov–Smirnov (KS) goodness of fit with 95% confidence level, K-distribution modelled data presented statistical differences from original raw data, showing that it is not an adequate fit for the analyzed problem.

Other study comparing K-distribution with Rayleigh and Gamma distributions was presented by Ge et al.^29^ in pig and mouse biological tissues. The K-distribution was the best fit for the excised pig brain (cortex) using a KS test (p value=0.943).

The two-parameter Weibull distribution^4^ can be obtained from GG distribution for d=p:

$$p_{W}(A;a,d)=\frac{dA^{d-1}}{a^{d}}e^{-{\left(A/a\right)}^{d}}.$$ (5)

Jesus et al. tested this distribution to model corneal speckle intensities.^4^ No statistically significant difference was observed between the fitted and the raw data (p value<0.05, KS goodness of fit test), concluding that the Weibull distribution can be used to model these data.

The Nakagami distribution is a two-parameter distribution that can be obtained from the Gamma distribution by setting a=Ω/d and taking the square root of the original random variable, $A^{\prime }=\sqrt{A}$:^30^

$$p_{NK}(A^{\prime };d,\Omega )=\frac{2d^{d}}{\Gamma (d)\Omega^{d}}A^{\prime 2d-1}e^{-\frac{d}{\Omega }A^{\prime 2}},$$ (6)

where d is a shape parameter and Ω is a spread parameter.

This distribution has been proposed to represent the dispersion of several backscattered clusters of incoherently added waves^15^ and has been tested for modeling skin speckle data against other distributions. It was considered the best fit, using the KS test, and its shape parameter presented higher values for epidermis, compared with stratum corneum, allowing for a threshold definition to separate both groups.

Nakagami distribution was also tested in corneal data.^4^^,^^25^ For this case, although it was not considered the best fit, fitted data did not present statistical significant differences from the raw data, also using the KS test.

The Rician, or Rice distribution [Eq. (7)] is a two-parameter generalization of the Rayleigh distribution [Eq. (1)], obtained by the introduction of a noncentrality parameter:

$$p_{\mathrm{RI}}(A;a,\nu )=\frac{A}{a^{2}}e^{-\frac{A^{2}+\nu^{2}}{2a^{2}}}I_{0}(\frac{A\nu }{a^{2}}),$$ (7)

where ν is the noncentrality parameter and $I_{0}$ is the zero-order modified Bessel function of the first kind.^31^ Thus, the Rayleigh can be obtained from the Rician distribution for ν=0.

The validity of this distribution for modeling speckle amplitude distribution was tested for tissue phantom data by Seevaratnam et al.^1^ and for corneal data, by Jesus et al.^4^ However, for the corneal data, the data modeled with this distribution presented statistically significant difference from raw data (KS test for a 95% confidence level). Nevertheless, the Rician distribution can be used to model speckle data when a dominant reflector, such as a tissue boundary, is present. In this particular case, the speckle is not fully developed, because the phases of the speckle field are not uniformly distributed in the interval (−π, π), having a bias accounted by the noncentrality parameter of the distribution (ν).^7^

The three-parameter Rayleigh distribution is obtained by modifying the Rayleigh [Eq. (1)] including two new parameters, b and c:

$$p_{3\mathrm{RL}}(A;a,b,c)=\frac{b(A-c)}{a^{2}}e^{-\frac{-{\left(A-c\right)}^{2}}{2a^{2}}},$$ (8)

where a is the scale parameter, b is the amplitude normalization parameter, and c is the shifting parameter. Matveev et al.^32^ and Demidov et al.^33^ used spatial speckle statistics on OCT lymphangiography and neurography to map lymphatic vessels, based on the analysis of the parameters of $p_{3\mathrm{RL}}$. Their experiments, on normal skin and tumor tissues, showed that, by fitting Eq. (8) to different regions of interest (ROIs) in an image, the obtained $R^{2}$ values statistically differed from each other, and could then be used as a feature for nerves and lymphatic vessels mapping. Using a threshold on the $R^{2}$ value ($0.9<R^{2}<0.99$ for the lymph vessels), the authors were able to obtain a discrimination of the tumor from the normal tissue. Following their previous studies, Matveev et al.^34^ presented an optimization model to automatically determine the threshold for the $R^{2}$ value and for the size of the ROI, both parameters previously empirically chosen.

The Lognormal distribution is a two-parameter distribution of a variable whose logarithm follows a normal distribution, with mean υ and standard deviation σ. Its PDF is given by Eq. (9) and can be derived from the GG distribution [Eq. (3)] by setting d/p→∞:

$$p_{L}(A;\mu ,\sigma )=\frac{1}{\sigma A\sqrt{2\pi }}e^{-\frac{{\left(\log\;A-\upsilon \right)}^{2}}{2\sigma^{2}}}.$$ (9)

The Lognormal distribution has been applied by Jesus et al.^4^ and Mcheik et al.,^15^ on corneal and skin speckle data, respectively. Both studies have been further described in Sec. 3.2.2, as the authors compare the Lognormal distribution with the GG distribution in both cases. They also obtain the same conclusion: the GG distribution is a better fit than the Lognormal for corneal and skin speckle data, and data modeled with Lognormal distribution show statistically significant difference from the original data, using the KS test with a level of significance of 0.05.

A form of the Burr type XII distribution was recently used to model OCT speckle data.^29^^,^^35^ It corresponds to a two-parameter distribution for non-negative random variables, where one of its shape parameters (c) is set to 2 and can be described as

$$p_{\mathrm{burr}}(A;b,\lambda )=\frac{2A(b-1)}{\lambda^{2}{\left[{\left(\frac{A}{\lambda }\right)}^{2}+1\right]}^{b}},$$ (10)

where b is the power law parameter and λ is the scale parameter. This distribution was used by Ge *et al.*^29^ to model speckle OCT data in a gelatin phantom with milk, and a set of *ex vivo* tissue: mouse brain, mouse liver, pig cornea, and chicken muscle. The Burr derived distribution achieved the best fit (KS tests), when compared with the Rayleigh, K, and gamma distributions for all the tissues. Finally, the authors also tested OCT data from an *in vivo* human hand’s palm and the Burr derived PDF achieved the best KS (p value=0.947).

#### 3.2.4 Nonparametric approach

Niemczyk and Iskander^36^ proposed a new nonparametric approach for the statistical analysis of OCT speckle amplitudes based on a comparison with a benchmark Rayleigh distribution ($a=\sqrt{2}/2$). First, the empirical cumulative distribution function, KDE, empirical characteristic function, and contrast ratio (CR), were determined in the region of interest of the OCT B-scans. Then, a set of four statistical distances between the empirical distribution and the benchmark distribution were determined to characterize the sample in study. This method was applied to resin phantoms with nine different concentrations of 10-μm blue dye powder particles, to *ex vivo* porcine eyes, and to *in vivo* human corneas with varying IOP values. The results showed that a better goodness-of-fit achieved with a particular parametric distribution does not necessarily correspond to a better discriminating power of the IOP.

**Fig. 6:**
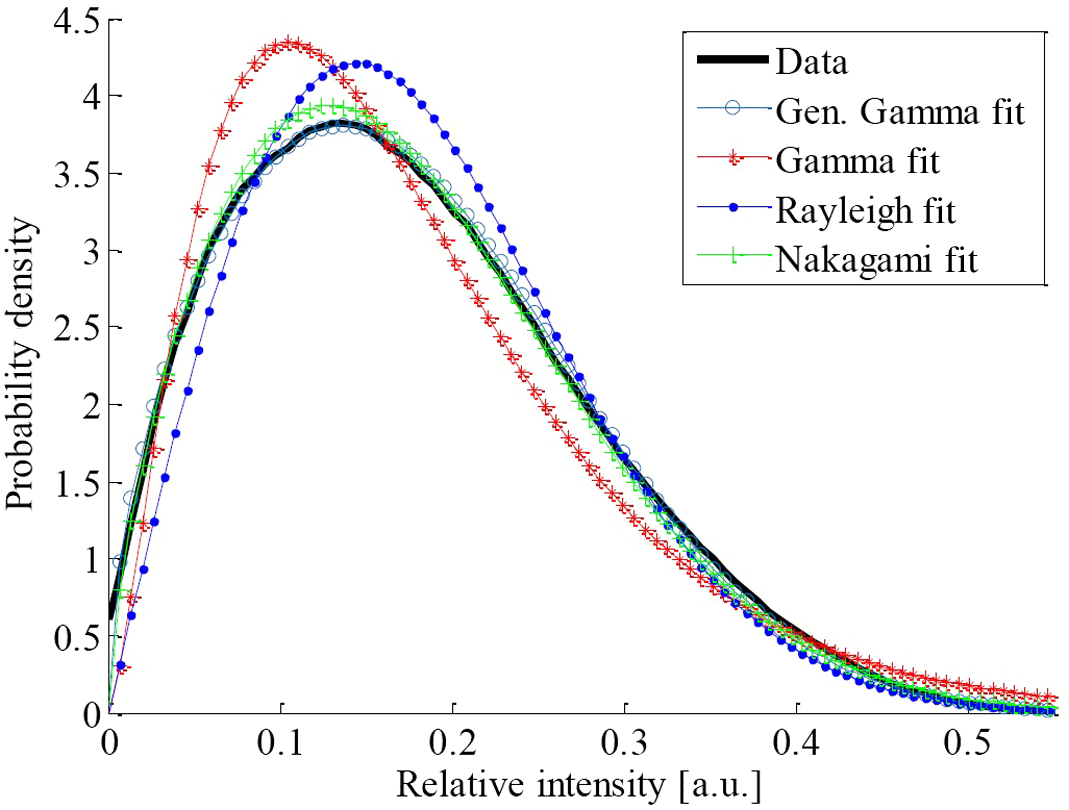
GG, Gamma, Rayleigh, and Nakagami distributions fit to speckle corneal data. Reproduced from Jesus et al.^25^ with the authors’ permission.

All distributions presented in this section have been proposed to model speckle images for different applications. The preferred distribution will vary depending on the case. For this reason, several authors opt to test different distributions to choose the better fit, based on a defined statistical criteria. Figure 6 shows an example of GG, Gamma, Rayleigh, and Nakagami functions applied to speckle data from the cornea.

#### 3.2.5 Statistical distribution in dynamics speckle analysis

The temporal speckle distribution of a single pixel is expected to follow different distributions according to the properties of the sample in that pixel. Therefore, a statistical distribution can be applied to the time-domain histogram of individual pixels to account for speckle dynamics.

Cheng et al.^37^ analyzed OCT voxels denoting fluid flow for large and small arterioles and venules in phantom and skin data. This analysis was performed as part of a visualization enhancement technique. The authors state that a pixel located within static tissue is expected to follow a Gaussian distribution over time, while pixels located in regions depicting flow will follow a different distribution. In their results, they concluded that Rayleigh distribution [Eq. (1)] is suitable to describe the speckle temporal distribution of large arterioles and venules, while the Rician distribution [Eq. (7)] can model the amplitude of OCT for tissues that are partially denoting static and flowing scatterers, such as capillaries.

### 3.3 Contrast Ratio

The CR (C) of an OCT image can be defined as the ratio of the signal’s standard deviation, (σ), and its mean, (μ):^38^

$$C=\frac{\sigma }{\mu }.$$ (11)

The CR is expected to vary with the density of scatterers and has been used for different applications, such as segmentation and motion estimation.

#### 3.3.1 Static analysis

Hillman et al.^38^ and Duncan et al.^39^ both proved that it is possible to obtain a correlation between the local contrast statistics and the scatterers density in an OCT sample. Hillman et al.^38^ theoretically demonstrated that the CR decreases with the increase of the effective number of scatterers that contribute to the signal. The authors also confirmed empirically their predictions using an experimental setup of controlled tissue phantoms of suspensions of microspheres in water with different concentrations. Duncan et al.^39^ computed the local contrast image of simulated synthetic speckle patterns. Then, they estimated the relation between the lognormal distribution [Eq. (9)] parameters of this image and the size of the image kernel. Experiments using chick embryo OCT images were also performed. Vessels and background were successfully segmented by choosing an adequate threshold of the lognormal PDF parameters computed in the contrast image.

#### 3.3.2 Dynamic speckle analysis

Kirkpatrick et al.^40^ developed an approach to quantify the shift and temporal contrast in a translating speckle pattern. The end goal of the authors was to quantify local motion. Their proposed method, quantitative temporal speckle contrast imaging, is dependent on the speckle size in the image and the number of images in a sequence. The application of the method is also depends on the acquisition speed of the device, which should be fast enough that no motion occurs during each image acquisition within the sequence.

An OCT optimized approach was also developed by Mariampillai et al.^41^ using only speckle local variance to image microvasculature in high and low bulk tissue motion scenarios. The speckle variance was computed using different number of frames (gate length), and a set of optimized parameters was determined for each tissue motion scenario. In the case of low bulk tissue motion (mice dorsal window chamber), the optimized gate length was defined in the range of 8 to 32 frames. For the high-bulk tissue motion (human nail root), the optimized gate length was two frames.

### 3.4 Logarithmic Pixel Intensity Contrasts

OCT images are often displayed in a logarithmic scale to enhance dynamic range. This transformation causes some properties, primary the contrast, to change when compared with OCT data in linear scale.

#### 3.4.1 Static analysis

Theoretically, when considering a set of OCT linear data where amplitudes follow the Rayleigh distribution [Eq. (1)] and have the same contrast value, its logarithmic transformation will result in a CR which depends on the signal intensity.^16^

Contrast changes in logarithmic scaled OCT data were experimentally illustrated by Lee and Zhang^16^ using phantoms made of intralipid solution and *in vitro* mouse livers images. They have determined that the logarithmic speckle contrast increases with the decreasing signal intensity. In addition, this fact was used to characterize the scattering properties of the studied phantoms using a method called depth-dependent speckle contrast. Since the mean OCT signal intensity decreases with the image depth but the tissue properties are relatively constant, the logarithmic contrast showed an increasing tendency for deeper areas. The magnitude of this slope was then related with the scattering properties of the phantoms.

#### 3.4.2 Dynamic speckle analysis

The logarithmic intensity variance (LOGIV) is defined as the variance of the intensity image after the logarithmic transformation. The differential logarithmic intensity variance (DLOGIV) is calculated by multiplying the LOGIV by a factor of two. LOGIV and DLOGIV values approach $\pi^{2}/6$ and $\pi^{2}/3$, respectively, when the signal-to-noise ratio approaches zero. These values can be used to compute LOGIV and DLOGIV tomograms by collecting multiple scans from the same region and measuring the quantitative variance of logarithmic intensities over scans. Motaghiannezam and Fraser^42^ proposed this technique for the analysis of *in vivo* human retinal vasculature visualization. Results of their experiments showed that static areas of the retina were invisible in the LOGIV and DLOGIV tomograms, while areas with detectable motion, such as blood vessels, were not. They also confirmed the low sensitivity of LOGIV and DLOGIV to the sample reflective strength, demonstrating the superiority of these methods in comparison to linear CRs for detecting motion and visualizing microvasculature.

### 3.5 Spatial Gray-Level Dependence Matrices

**Fig. 7:**
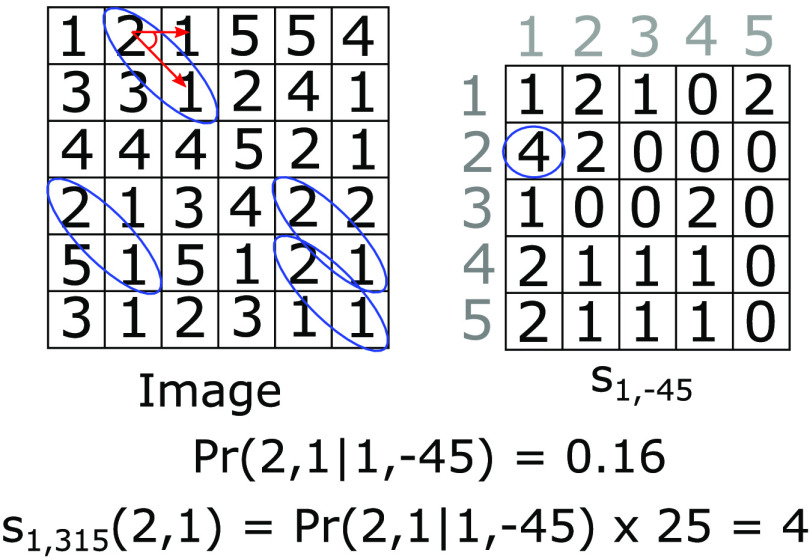
Diagram representing the SGLDM for a direction of θ=−45  deg and distance of d=1. The image has N=25  pixels with levels between 1 and 5. The blue ellipses indicate the number of pairs (2,1) on the specified direction and distance.

Spatial gray-level dependence matrices (SGLDM), also referred to as co-ocurrence matrices,^2^^,^^8^^,^^43^^,^^44^ are determined by the estimation of the second-order joint probability distribution of two image gray levels which are at a specific distance and direction (Pr(i,j|d,θ)). Assuming the images are normalized with L grayscale levels and N number of pixels, each Pr(i,j|d,θ) is the probability of a pixel with level i being at a distance d from a pixel with gray level of j in the θ direction. Following this principle, an L×L co-occurrence matrix can be created for each direction and for each chosen distance $s_{\theta ,d}(i,j)=\mathrm{Pr}(i,j|d,\theta )\times N$, as is shown in Fig. 7.

Several features can be extracted from these matrices, including energy, entropy, correlation, local homogeneity, and inertia, detailed in Refs. 2, 43, 44. Similarly to the statistical properties described in Sec. 3.1, these features are expected to be linked to changes in tissue. Thus, they can be used for classification tasks.

Kasaragod et al.^8^ used SGLDM to retrieve information from the speckle OCT images. A Bayesian model was applied to the classification of tissue phantoms with different amount of scatterers and to identify the invasion of melanoma cell into tissue engineered skin. Their results were satisfactory in classifying the number of scatterers in the tissue phantoms, shown visually by an ROC curve plot. However, this approach provided limited results in the identification of the melanoma cells in the tissue. Nevertheless, sensitivity and specificity numerical results were not provided in the paper.

### 3.6 Frequency Domain Methods

After computing the two-dimensional (2D) discrete Fourier transform (DFT) in an OCT image, the resulting image can be divided into regions, according to their frequency content. This will result in different texture parameters per region. The contribution of each region to the total frequency magnitude is calculated by summing all the values of the spatial frequencies in that region and dividing by the total frequency magnitude of the image. This value represents the percentage of signal within a certain range of spatial frequencies, and it can be used as a feature in similar applications as described in Secs. 3.1 and 3.4.

Gossage et al.^2^^,^^43^ used the 2D-DFT for retrieving information from OCT images, together with the previously described SGLDM (Sec. 3.5). The goal of the work was analyzing and classifying texture of different tissues (mouse and bovine tissue). Their results showed a high accuracy classification of mouse skin and fat, of 98.5 and 97.3%, respectively. A satisfactory performance was also obtained for distinguishing normal and abnormal mouse lung, of 64.0 and 88.6%, respectively. Finally, the features were used to classify five different bovine tissues, with a similar classification model, resulting in an average of 80% correct classification rate. A similar approach was used in Ref. 44, where the 2D-DFT and SGLDM features were used to differentiate living from nonliving tissue phantoms with various sizes and distributions of scatterers.

### 3.7 Tissue Dispersion

Tissue dispersion can be measured in a static image analysis from the degradation of the image point spread function (PSF). The standard method to measure tissue dispersion is through the computation of the resolution degradation of single reflections. However, such a measurement requires distinct point reflectors, below and outside the sample, which rarely happens *in vivo*.

As alternative, Photiou et al.^45^^,^^46^ proposed to estimate the tissue dispersion from the imaged speckle. Being a coherent phenomenon, speckle is affected by tissue dispersion. Changes in speckle size can be used to estimate the broadening of the image PSF and later to calculate the group velocity dispersion (GVD). This method is based on the comparison of small regions of an OCT image at different depths without visible structures, i.e., containing only speckle information. When the OCT interferometer arms imbalance is removed at the tissue surface, it is expected that these surface regions show no dispersion, as opposed to deeper regions.

Photiou et al.^45^^,^^46^ showed that their proposed method performs similarly to the standard procedure (from degradation of the image PSF), with a GVD difference less than 7%. Also, the median of the GVD proved to be a good feature for tissue classification, when comparing normal (higher median) to malignant (lower median) samples of human colon (accuracy of 96% using linear discriminant analysis).

### 3.8 Speckle Correlation

Considering a sequence of scans over time, the autocorrelation and/or decorrelation of intensities can carry information about motion of particles in a sample. Both features can be used as an approach for time-varying speckle analysis.^6^

In terms of speckle analysis, the normalized autocorrelation, for a given point p and time lag τ, is given as

$$g_{p,\tau }=\frac{\overset{N-1}{\underset{t=0}{\sum }}(I_{p,t}-\langle I_{p}\rangle )(I_{p,t+\tau }-\langle I_{p}\rangle )}{\overset{N-1}{\underset{t=0}{\sum }}{\left(I_{p,t}-\langle I_{p}\rangle \right)}^{2}},$$ (12)

where $I_{p,t}$ is the intensity in the point p at time t, τ is the period of time between scans, and N is the total number of scans considered. The temporal average intensity, $\left\langle I_{p}\right\rangle$, is subtracted from each intensity value to consider only the intensity fluctuations. The autocorrelation of a speckle pattern with itself (τ=0) is expected to be maximized. With the increase of the lag between scans, this value is expected to decrease until it reaches 0, when the scans are no longer correlated. A vector of autocorrelation values can be obtained by changing τ. These autocorrelation values are related with the fluctuations in the intensity of the speckle patterns. At the same time, the fluctuations are related with the flow of particles in the tissue.^6^

The decorrelation time $\tau_{c}$ is used to analyze the shape of each autocorrelation function.^6^ This metric corresponds to the time it takes for the autocorrelation value to fall to 1/e.

#### 3.8.1 Temporal analysis

De Pretto et al.^6^ performed experiments with milk pumped through a microchannel at different velocities and proved the inversely proportional relation between decorrelation time and flow velocity. As expected, this relation is highly dependent on the sampling frequency. A sampling rate of 8k Hz makes the system appropriate for differentiating between low flow rates, up to 12  μl/min. However, if the flow rates are higher, the temporal resolution of the system makes it unsuitable.

In a later study, De Pretto et al.^47^ implemented the same approach to monitor blood sugar in OCT data. They used samples of heparinized mouse blood, phosphate buffer saline, and different concentration of glucose. They were able to differentiate between low level of glucose concentration, up to 355 mg/dL, indicating the suitability of OCT for noninvasive measurements of glucose levels.

Farhat et al.^48^ assessed the changes that occur in intracellular motion as cells undergo apoptosis. To that end, they induced apoptosis in samples of acute myeloid leukemia cells, and they measured the decorrelation time of the speckle over a period of 48 h. Their results showed an increase of motion in the cells (identified as a decrease of the decorrelation time of speckle) after 24 h, which is in accordance with histology. Ferris et al.^49^ used phantom data to study the effects of multiple scattering on the speckle decorrelation. Their conclusions confirm that speckle decorrelation is dependent on parameters such as the concentration and size of particles and velocity field inhomogeneities. They also concluded that an overestimation of blood flow velocities might occur because an increase in the rate of decorrelation is caused by the detection of forwardscattered light.

#### 3.8.2 Spatial and angular analysis

Popov et al.^50^ conducted an experiment using tissue phantoms. They obtained an expression for the spatiotemporal correlation function of scattered radiation, assuming a single scatterer regime, and were able to measure the viscosity of a solution with different concentrations of glucose with 1% error in stagnant conditions and 4% to 10% error in flow experiments.

Uribe-Patarroyo et al.^51^ proposed a new discrete normalized second-order autocorrelation. The authors claim this approach is more robust to the presence of noise and can be used as a method for speed measurements in tissue phantoms. This same method was used in a later work^52^ for the correction of the rotation distortion in catheter-based endoscopic OCT.

Liu et al.^53^ used the cross-correlation coefficient between adjacent A-scans to analyze motion speed of simulated speckle images. Their results underline the importance of computing speckle features, such as the contrast and decorrelation time, in larger sets of A-scans to achieve a more accurate estimation of the tissue properties. Due to the random nature of speckle, when a small set of A-scans is used, only local metrics are determined, resulting in larger errors when compared with the theoretically expected value.

Finally, speckle cross-correlation between OCT B-scans over a range of increasingly overlapping detection angles was used by Hillman et al.^54^ to quantify the singly scattered contribution to the speckle signal. They have used phantoms with lower and higher scatterers concentrations to quantity the prevalence of signal-carrying speckle at a specific depth and, therefore, the level of image fidelity to the underlying scatterers’ distribution.

## 4 Discussion

In most of the reviewed works, the authors aimed to further understand the physical meaning of the light speckle and how to model it mathematically.^3^^,^^16^^,^^17^^,^^21^^,^^39^^,^^40^^,^^49^^,^^53^^,^^55^ Although its interpretability remains a challenging task, a number of works have shown the feasibility of speckle-derived quantifications for biomedical imaging related tasks, including classification (e.g., healthy/pathological),^1^^,^^2^^,^^4^^,^^8^^,^^12^^–^^14^^,^^23^^–^^26^^,^^32^^–^^34^^,^^37^^,^^43^^–^^46^ segmentation (e.g., vessels),^15^ motion quantification (when dynamic data is provided),^6^^,^^48^^,^^49^^,^^51^^–^^53^ and image fidelity.^54^ Nevertheless, some aspects and limitations of those techniques should be discussed to improve future works.

Among all the reviewed methodologies, speckle modeling using statistical distributions has been the most studied, especially on static data. Several authors have proven the applicability of these approaches to different tasks, mainly to classify between different tissues. However, the conclusions on the optimal PDF often vary depending on the analyzed tissue, the application, and the OCT device. For example, Mcheik et al.^15^ and De Jesus et al.^4^ both presented a comparative study including several PDFs, but in different applications, one to differentiate speckle from different skin layers, and the second from different groups in corneal data. Despite both works including Nakagami, GG, and Lognormal distributions, their conclusions were different. For skin layers, the Nakagami distribution was found to be the best. However, for corneal data, the best fit was achieved by the GG distribution. Furthermore, other authors have successfully applied other distributions to the same application, such as Niemczyk et al.,^23^ who used the Gamma distribution to study the effect of IOP on corneal OCT speckle from porcine eyes. In addition, a recently used distribution (Burr) seems promising in modeling OCT speckle amplitude in several tissues (e.g., mouse, pig, chicken, and human^29^) and can also be used to the study of IOP.^36^ The lack of strong evidence provided by the studies precludes drawing conclusions on the PDF which provides the best fit for each application, since in some of them, exhaustive comparisons and physical meaning do not exist. In this line, a new nonparametric approach^36^ was proposed as a way to reduce the complexity of speckle statistical modeling, since there is no need to select a particular PDF.

A drawback that the modeling of speckle through statistical distributions had to tackle is that the real-world problems do not fulfill the theoretical assumptions of speckle formation. Theoretically speaking, when the number of elementary phasors is high, meaning a high number of scatterers per coherence volume, the central limit theorem is fulfilled,^16^ the speckle pattern is fully developed, and its intensity distribution follows the Rayleigh distribution. However, this argument is only partially applicable to biological tissues, because different tissues may have different natures, i.e., some can be more heterogeneous with a lower number of scatterers.^33^ As a consequence, several authors propose more complex distributions (three parameters and higher), which presents new potential challenges. When a statistical model is used to represent the process that generated the data, the representation will not be fully accurate, as some information will be lost. In estimating the amount of information lost by a model, one needs to take into account the trade-off between the goodness of fit and the simplicity of the model, i.e., the risk of overfitting and underfitting. In the corneal studies,^4^^,^^25^^,^^26^ the Akaike’s information criterion is applied to minimize this risk.

Following the analysis of statistical distributions, the second most used technique is the analysis of the speckle correlation, which is only applicable to dynamic data or spatially/angularly diverse data. The main application of this technique is in motion determination (e.g., blood flow). Although most of the reviewed works in OCT speckle are focused on theoretical modeling or validation with phantoms, this is in fact a type of signal analysis that has been widely explored in other imaging modalities. Some examples are laser speckle imaging, which can be used for cutaneous blood flow determination,^5^^,^^56^ blood pulse pressure waveform estimation,^57^^,^^58^ cellular assessment in muscle tissue,^59^ and laser speckle flowgraphy which can be used for ocular blood flow determination.^60^ Furthermore, the rationale behind these techniques has also been widely applied to compute OCT angiography from a set of temporal OCT data acquired at the exact same position.^61^

In contrast with the correlation analysis, there have been little to none applications of the other methods for dynamic data. However, some authors have demonstrated the feasibility of statistical properties,^14^ statistical distributions,^37^ or logarithmic intensity contrast^42^ to visualize and segment blood vessels within a tissue.

Finally, the methods that compute characteristics of static data [tissue dispersion, SGLDM, frequency domain methods, or the previously mentioned statistical properties/distributions and contrast (both with and without the application of the logarithmic transform)] are more applied to obtain features to use in a predictive model or classifier. While these approaches may underperform classification models that take into account the complete image information or several features, many of these proposed features are very easy to interpret, and to link to the physical changes in the tissue, making them interesting for clinical practice.

Although a growing interest in the analysis of signal-carrying speckle has been observed over the last years, it is still a research line in an early stage. A considerable number of different methods have already been proposed, but there are only a few applications published in the literature. At the current stage, the analysis of OCT speckle is lacking on validation and information on its reproducibility *in vivo*. None of the reviewed studies validated the proposed methods on large (the largest dataset in the studies included had 65 subjects^4^^,^^25^) or multiple datasets. The speckle information is intrinsically related to the spatial arrangement and biomechanical properties of the scatterers in the sample. Scatterers can either be collagen fibers and fibroblasts when imaging the cornea, blood cells flowing through vessels, or just silica particles in a phantom image. What is considered a scatterer in a sample will depend on the relation between the characteristics of the imaging system, namely beam spot size, coherence gate^38^ and light source wavelength, and the particles size and concentration.^62^ This is particularly important for OCT imaging, given the variability existing between devices (790 to 1330 nm), as it can be observed in Table 1, Appendix A. Consequently, for the same sample and method, different quantitative values for speckle may be obtained depending on the OCT system used.

Another important aspect that hampers the development of speckle-based techniques is the limited access to raw data. Images collected from commercial OCT devices are often filtered to reduce the speckle or transformed to increase visualization contrast. It is of utmost importance that raw OCT images are used in speckle studies, otherwise the obtained results are tainted by the used device and preprocessing algorithm.^63^ If raw data are not available, the information of the applied image processing algorithms should be provided to understand how the speckle has been processed and hence, comprehend its physical meaning in a biomedical application.

Despite its early stage, research on methods to study the signal-carrying speckle has been a step forward on the comprehension of the physical meaning of the information retrieved from OCT imaging. Speckle analysis provides information on the size and distribution of the scatters that has not been considered in a clinical practice yet. Such advancements are also particularly interesting for other research lines, such as OCT elastography, adaptive optics imaging, or machine learning applications. For example, recent developments on machine learning, namely on convolutional neural networks, have reported outperforming results in OCT image analysis in comparison to conventional image processing.^64^^–^^66^ However, deep learning approaches still lack on interpretability and roughly remain a black box, despite the recent efforts to address this limitation.^67^ Therefore, future research may focus on integrating physics and learning based approaches, to combine their strengths.

## 5 Conclusion

This paper presents an overview of the current state of the art in OCT signal-carrying speckle analysis in biomedical applications. The results of this literature review show that several methods have already been proposed for different applications, highlighting the potential of speckle analysis to infer the optical and spatial properties of the scatterers in a sample or tissue. However, signal-carrying speckle analysis in OCT is still in its early stage, and further work is needed to validate its applicability and reproducibility in a clinical context.

## 6 Appendix A. Article Details

Characteristics of the reviewed studies sorted by type of method are shown in Table 1.

**Table 1:** Characteristics of the reviewed studies. Articles sorted by type of method.

Method	Static/dynamic	Authors and publication year	Aim	Application/data used	OCT technique (brand)	Light wavelength (nm)
Statistical properties	Static	Wang et al.^13^ (2013)	Classification	*Ex vivo* human tissue	SS-OCT (custom made)	1310
Statistical properties	Static	Roy et al.^12^ (2015)	Classification	Coronary artery	SD-OCT (CV-M2, LightLab Imaging Inc.)	1320
Statistical properties	Dynamic	Ossowski et al.^14^ (2015)	Classification	Blood	SD-OCT (custom made)	790
Statistical distributions	Static	Schmitt et al.^3^ (1999)	Theoretical modeling	—	—	—
Statistical distributions	Static	Karamata et al.^55^ (2005)	Theoretical modeling	—	—	—
Statistical distributions	Static	Mcheik et al.^15^ (2008)	Segmentation	Skin	SD-OCT (SkinDex 300, ISIS)	1300
Statistical distributions	Static	Kirillin et al.^21^ (2014)	Theoretical modeling	Tissue phantoms (polystyrene microspheres)	SS-OCT (custom made)	1310
Statistical distributions	Static	Seevaratnam et al.^1^ (2014)	Classification	Tissue phantoms (polystyrene microspheres)	SS-OCT (Biophotonics and Bioengineering Laboratory’s)	1310
Statistical distributions	Static	Jesus et al.^25^ (2015)	Classification	Cornea	SD-OCT (Copernicus HR)	850
Statistical distributions	Static	Almasian et al.^17^ (2017)	Theoretical modeling	Tissue phantoms (silica microspheres)	SS-OCT (Santec IVS 2000)	1309
Statistical distributions	Static	Jesus et al.^4^ (2017)	Classification	Cornea	SD-OCT (IOLMaster 700)	850
Statistical distributions	Static	Jesus et al.^24^ (2017)	Classification	Cornea	SD-OCT (Copernicus HR)	851
Statistical distributions	Static	Demidov et al.^33^ (2019)	Classification	Mice (skin)	SS-OCT (custom made)	1320
Statistical distributions	Static	Matveev et al.^32^ (2019)	Classification	Mice (skin)	SS-OCT (custom made)	1320
Statistical distributions	Static	Matveev et al.^34^ (2019)	Classification	—	—	—
Statistical distributions	Static	Iskander et al.^26^ (2020)	Classification	Cornea	SD-OCT (HRT 3, Heidelberg Engineering GmbH)	850
Statistical distributions	Static	Niemczyk et al.^23^ (2021)	Classification	Cornea (porcine eyes)	SD-OCT (Copernicus REVO)	830
Statistical distributions	Static	Danielewska et al.^28^ (2021)	Classification	Cornea (rabbit eyes)	SD-OCT (Copernicus HR)	850
Statistical distributions	Static	Ge et al.^29^ (2021)	Classification	Phantom, mouse (brain/liver), pig (brain/cornea), chicken muscle, skin	SS-OCT (custom made)	1310
Statistical distributions	Static	Niemczyk and Iskander^36^	Classification	Phantom, cornea (porcine and eyes)	SD-OCT (Copernicus REVO)	830
Statistical distributions	Dynamic	Cheng et al.^37^ (2014)	Classification	Phantom: agrose and titanium dioxide/skin	SS-OCT (Thorlabs Inc.)	1300
Tissue dispersion	Static	Photiou et al.^45^ (2017)	Classification	Porcine muscle/adipose tissues/colon	SS-OCT (custom made)	—
Tissue dispersion	Static	Photiou et al.^46^ (2017)	Classification	Porcine muscle/adipose tissues/colon	SS-OCT (custom made)	1300
SGLDM	Static	Kasaragod et al.^8^ (2010)	Classification	Tissue phantoms (agar intralipid solution)/tissue engineered (skin)	SS-OCT (custom made)	1315
SGLDM/frequency domain methods	Static	Gossage et al.^2^ (2003)	Classification	Mouse lung	SS-OCT (custom made)	1300
SGLDM/frequency domain methods	Static	Gossage et al.^43^ (2003)	Classification	Mouse lung/bovine tissues	SS-OCT (custom made)	1300
SGLDM/frequency domain methods	Static	Gossage et al.^44^ (2006)	Classification	Tissue phantoms (silica microspheres)/bovine aorta endothelial cells	SS-OCT (custom made)	1300
CR	Static	Hillman et al.^38^ (2006)	Theoretical modeling	Tissue phantoms (polystyrene microspheres)	SD-OCT (custom made)	1330
CR	Static	Duncan et al.^39^ (2008)	Theoretical modeling/segmentation	Embryonic chick heart	—	—
CR	Dynamic	Kirkpatrick et al.^40^ (2007)	Theoretical modeling/motion determination	Engineered tissue	SD-OCT (custom made)	843
Logarithmic intensity contrasts	Static	Lee et al.^16^ (2011)	Theoretical modeling	Rat liver/tissue phantoms	SD-OCT (custom made)	834
Logarithmic intensity contrasts	Dynamic	Motaghiannezam and Fraser^42^ (2012)	Visualization	Retina	SS-OCT (custom made)	1060
Speckle correlation	Dynamic	Farhat et al.^48^ (2011)	Motion determination	Acute myeloid leukemia cells	SS-OCT (Thorlabs Inc.)	1300
Speckle correlation	Dynamic	Liu et al.^53^ (2013)	Motion determination	—	—	—
Speckle correlation	Dynamic	Uribe-Patarroyo et al.^51^ (2014)	Motion determination	Tissue phantoms (intralipid)	SS-OCT (custom made)	1285
Speckle correlation	Dynamic	De Pretto et al.^6^ (2015)	Motion determination	Milk flow	SS-OCT (Thorlabs Inc.)	1325
Speckle correlation	Dynamic	Uribe-Patarroyo et al.^52^ (2015)	Motion determination	Endoscopic (esophagus)	SD-OCT (NvisionVLE)	1310
Speckle correlation	Dynamic	De Pretto et al.^47^ (2016)	Viscosity determination	Mice blood	SR-OCT (Thorlabs Inc.)/ SS-OCT (custom made)	930 /1325
Speckle correlation	Dynamic	Popov et al.^50^ (2017)	Viscosity determination	Tissue phantoms	SD-OCT (custom made)	1313
Speckle correlation	Dynamic	Ferris et al.^49^ (2020)	Motion determination	Tissue phantoms	SD-OCT (custom made)	1290 /1310

SGLDM, spatial gray level dependence matrices; SD, spectral domain; SS, swept source; SR, spectral radar; NvisionVLE, NvisionVLE imaging system (NinePoint Medical, Inc., Bedford, Massachusetts); Thorlabs Inc., Thorlabs Inc. (Newton, New Jersey); IOLMaster 700, IOLMaster 700 (Carl Zeiss Meditec AG, Germany); CV-M2, LightLab Imaging Inc., CV-M2, LightLab Imaging Inc. (Westford, Massachusetts); Copernicus HR, Copernicus HR (Optopol, Zawiercie, Poland); HRT 3, Heidelberg, HRT 3, Heidelberg Engineering GmbH (Heidelberg, Germany); Copernicus REVO, Copernicus REVO, (Optopol, Zawiercie, Poland).

## 7 Appendix B. Speckle Theory

As detailed in the paper, one of the main speckle analysis techniques is based on the determination of the speckle pattern intensity PDF, since photodetectors measure light intensity and not complex amplitude. A short theoretical introduction on the speckle effect is mandatory to understand why the negative exponential function was historically the first one used to describe the speckle statistics and to understand their limitations.

Light speckle is often modeled using the statistical perspective defined by J. W. Goodman.^68^ To deduce the PDFs of the speckle signal amplitude (Gaussian) and its respective intensity (negative exponential), we shall follow Goodman’s approach.

Assuming a monochromatic and perfectly polarized light source, for a given temporal instant, we can define the complex amplitude of the electrical field a as^69^

$$a(x,y,z)=a(x,y,z)e^{i\theta (x,y,z)},$$ (13)

where a(x,y,z) is the amplitude and θ is the phase. Regardless of the detector spatial position, the amplitude of the electrical field that reaches the detector A corresponds to a sum of N dephased electrical fields coming from different regions of the tissue:

$$A(x,y,z)=\frac{1}{\sqrt{N}}\sum_{k=1}^{N}a_{k}e^{i\theta_{k}}.$$ (14)

where $a_{k}$ and $\theta_{k},k=1,2,\ldots N$ are the amplitudes and phases forming that field. To determine the PDF of the complex amplitude, two assumptions related to the physical mechanisms of speckle are made. First, the amplitude and phase of each phasor are statistically independent of each other. Second, the phases $\theta_{k}$ are uniformly distributed between −π and π. In terms of physical significance, these assumptions imply that each scattering volume is independent and that the reflection boundary irregularities are larger than the light wavelength.

By splitting the complex amplitude in its real and imaginary parts, it can be shown that both have zero mean and identical variances.^68^ When the number of summed phasors is very large (N→∞), the PDF of the real and imaginary part are asymptotically Gaussian as well as their joint PDF:

p(ARe,AIm)=p(ARe)·p(AIm)=1σ22πe−ARe2+AIm22σ2, (15)

where ARe corresponds to the real part and AIm to the imaginary part of A(x,y,z), and the variance $\sigma^{2}$ is defined as

$$\sigma^{2}=\underset{N\to \infty }{\lim}\;\frac{1}{N}\overset{N}{\underset{k=1}{\sum }}\frac{\left\langle a_{k}^{2}\right\rangle }{2}.$$ (16)

The PDF of the light intensity can also be deduced from Eq. (15). By definition, the light intensity I and the phase θ are expressed as

I=|A(x,y,z)|2=ARe2+AIm2, (17)

θ=tan−1 AImARe. (18)

The relation between the intensity PDF and the amplitude PDF can be found by applying random variables transformations:

p(I,θ)=p(ARe,AIm)‖J‖=1σ24πe−I2σ2, (19)

where ‖J‖ is the Jacobian matrix. Recalling the assumptions of independence between intensity and phase, the marginal PDF of the intensity is found using

$$p(I)=\int_{-\pi }^{\pi }p(I,\theta )d\theta =\frac{1}{2\sigma^{2}}e^{-\frac{I}{2\sigma^{2}}}.$$ (20)

By computing the first- and second-order moments of the amplitude, the field amplitude variance can be expressed as^69^

$$\sigma^{2}=\langle I\rangle /2,$$ (21)

resulting in a negative exponential PDF, which is characteristic of a fully developed speckle pattern in perfect conditions:^3^

$$p(I)=\frac{1}{\left\langle I\right\rangle }e^{-\frac{I}{\left\langle I\right\rangle }}.$$ (22)
